# HAVE I GOT A JOB FOR YOU: OCCUPATION-RELATED ASTHMA THROUGHOUT LIFE IN THE UNITED STATES

**DOI:** 10.1093/geroni/igz038.1033

**Published:** 2019-11-08

**Authors:** Sarah B Laditka, James N Laditka, Ahmed Arif, Jessica Hoyle

**Affiliations:** University of Nortth Carolina at Charlotte, Charlotte, North Carolina, United States

## Abstract

Work exposures to asthma triggers can cause or aggravate asthma, which affects twenty-five million Americans including many older workers, and retirees who want to work or need to do so for income. Asthma trigger exposures have particular risk for older workers. Older adults who develop asthma have poorer health outcomes than people who had childhood asthma, yet older workers with low incomes may have limited ability to leave a job despite health risks. We studied occupation-related asthma using the nationally representative Panel Study of Income Dynamics (PSID) (1968-2015, n=13,957, 205,498 person-years). We compared asthma outcomes in occupations with likely asthma trigger exposures to those in occupations with limited exposures. Methods included: prevalence ratios; incidence risk ratios (log-binomial regression adjusted for age, sex, race/ethnicity, education, atopy, current and past smoking, and survey design); attributable risk fractions; population attributable risks; and microsimulation. The adjusted prevalence ratio comparing high risk occupations to low was 4.1 (95% confidence interval, CI 3.5-4.8); adjusted risk ratio 2.6 (CI 1.8-3.9); attributable risk 16.7% (CI 8.5-23.6); population attributable risk 11.3% (CI 5.0-17.2). In microsimulations, 14.9% (CI 13.4-16.3) with low trigger exposures reported asthma during working life, compared with 23.9% (CI 22.3-26.0) with high exposures. Asthma triggers at work may cause or aggravate more than 10% of adult asthma, and increase asthma risk by 60%. Lung health contributes importantly to well-being, and the ability to work at older ages. Results highlight needs for policies and employer actions to reduce asthma trigger exposures, and for public education about lung health.

